# Editorial: Assessment and management of adverse drug reactions in oncology

**DOI:** 10.3389/falgy.2023.1223078

**Published:** 2023-07-13

**Authors:** Ping-Feng Tsai, Kevin Sheng-Kai Ma

**Affiliations:** ^1^ Department of Ophthalmology, Tri-Service General Hospital, Taipei, Taiwan; ^2^ Division of Pharamacoepidemiology and Pharmacoeconomics, Department of Medicine, Brigham and Women’s Hospital, Harvard Medical School, Boston, MA, United States; ^3^ Department of Dermatology, Massachusetts General Hospital, Harvard Medical School, Boston, MA, United States; ^4^ Center for Global Health, Perelman School of Medicine, University of Pennsylvania, Philadelphia, PA, United States; ^5^ Department of Epidemiology, Harvard T.H. Chan School of Public Health, Boston, MA, United States

**Keywords:** immune checkpoint inhibitor, immune-related adverse event, Stevens-Johnson syndrome, opportunistic infection, corticosteroid

**Editorial on the Research Topic** Assessment and management of adverse drug reactions in oncology

## Introduction

New drugs such as tyrosine kinase inhibitors, monoclonal antibodies, angiogenesis inhibitors, and immune checkpoint inhibitors (ICIs) have been approved by the United States Food and Drug Administration for a variety of cancer types in recent years. Despite their remarkable success in overall survival and progression-free survival in clinical trials, a substantial proportion of patients suffer from severe or fatal adverse drug reactions (1, 2). Due to the diverse manifestations of adverse events associated with anti-neoplastic agents, the assessment and management of these adverse events remain largely unknown. Among all, the assessment and management of adverse events following the use of ICIs, also known as immune-related adverse events (irAEs), is an emerging field.

## Clinical presentations of irAEs

Contemporary guidelines including the Common Terminology Criteria for Adverse Events version 5.0 by the American Society of Clinical Oncology and the European Society for Medical Oncology provided the definition and grading system of organs commonly involved in irAEs, which facilitated the diagnoses of ICI-associated complications (Table 1) (3, 4). Organs commonly involved in irAEs include the skin, gastrointestinal tract, endocrine organs, and lungs; on the other hand, rare but fatal adverse events including neurotoxicity, cardiotoxicity, and pulmonary toxicity of ICIs have also been reported (3). Cutaneous toxicity is the most reported irAE. Reported manifestations of cutaneous irAEs include rashes, bullous dermatoses and severe cutaneous adverse reaction such as Stevens-Johnson syndrome/toxic epidermal necrolysis (SJS/TEN) and drug reaction with eosinophilia and systemic symptom (DRESS). Other common manifestations of irAEs include colitis, hepatitis, nephritis, thyroiditis, hypophysitis. The incidence of cardiovascular irAEs, such as myocarditis, pericarditis and vasculitis, was generally rare yet potentially fatal (5).

**Table 1:** Grading system and menifestations of common immune-related adverse events.

The organ(s)	Grade 1	Grade 2	Grade 3	Grade 4
Cutaneous Toxicity	- Target lesions covering <10% BSA and not associated with skin tenderness	- Target lesions covering 10%–30% BSA and associated with skin tenderness	- Target lesions covering >30% BSA - Severe/life-threatening symptoms - Generalized exfoliative/ulcerated/bullous rash	
Colitis	- Increase of fewer than 4 stools per day	- Mucus or blood in stool - Increase of 4 to 6 stools per day	Increase of 7 stools per day; incontinence; limiting self-care ADL	- Life-threatening consequences
Hepatitis (AST, ALT increased)	<3.0 × ULN - Asymptomatic	3.0–5.0 × ULN - Asymptomatic	5.0–20.0 × ULN - Symptomatic liver dysfunction - Compensated cirrhosis - Reactivation of chronic hepatitis	>20.0 × ULN - Decompensated liver function (ascites, coagulopathy, encephalopathy, coma)
Pneumonitis	- Asymptomatic - Confined to one lobe of the lung or <25% of lung parenchyma	- Symptomatic (dyspnea, cough or chest pain) - More than one lobe of the lung or 25%–50% of lung parenchyma	- Severe symptoms (new or worsening hypoxia) - All lung lobes or >50% of lung parenchyma - Need oxygen therapy	- Life-threatening respiratory compromise (need intubation and ventilator care)
Hypophysitis	- Asymptomatic or mild symptoms	- Moderate symptoms limiting age-appropriate instrumental ADL	- Severe or medically significant limiting self-care ADL	- Life-threatening consequences (visual field impairment)
Acute kidney injury (Cr increased)	1.0–1.5 × ULN, < 1.5 × baseline	1.5–3.0 × ULN, 1.5–3.0 × baseline	3.0–6.0 × ULN, > 3.0 × baseline	>6.0 × ULN, Dialysis indicated
Myasthenia gravis	- Asymptomatic or mild symptoms	- Moderate symptoms - Limiting age-appropriate instrumental ADL	- Severe or medically significant - Limiting self-care ADL	- Life-threatening consequences (respiratory muscle involved)
Myocarditis	- Asymptomatic - Cardiac enzyme elevation or abnormal EKG	- Symptoms with moderate activity or exertion	- Severe with symptoms at rest or with minimal activity or exertion	- Life-threatening consequences (hemodynamic impairment)

BSA, body surface area; ADL, activities of daily living; AST, aspartate aminotransferase; ALT, alanine aminotrasferase; ULN, upper limit normal; Cr, creatinine; EKG, electrocardiogram.

Several populations are at particular high risk of irAE, which include patients receiving ICI combination therapy; for instance, more frequent and more severe cardiovascular irAEs are observed in those treated with ICI combination therapies (5). In addition, patients with pre-existing autoimmune diseases are reported to be at great risk of irAEs or complications of irAE; likewise, given the underlying mechanisms of irAEs that involve self-reactive T cells, molecular mimicry, and antigen spread, and decreased immune tolerance (3), irAE in patients on ICI may resemble autoimmune flares (6).

Infections are commonly seen in patients with irAEs. As interfering with normal immune responses that can lead to opportunistic infections, studies have proposed that ICI-induced dysregulated immunity can lead to opportunistic infections such as Mycobacterium tuberculosis (TB) infections; that said, the consensus statement by the European Society of Clinical Microbiology and Infectious Diseases suggested undetermined causation, in which it was suspected that it was immunosuppressive treatments rather than ICIs that caused the infections (7). As the clinical presentation of irAEs is versatile and the pathogenic mechanisms remained unclear, regular monitoring of end-organ function in patients undergoing anti-neoplastic treatments would benefit their overall survival and quality of life.

## Diagnosis and assessment of irAEs

Drug causality assessment tools such as the algorithm of drug causality for epidermal necrolysis (ALDEN) or the Naranjo score can be used to classify the relationship between drug exposure and adverse drug reactions as ‘unlikely’, ‘possible’, ‘probable’, or ‘definite’ for severe cutaneous adverse reactions such as SJS/TEN (8, 9). However, there is currently no consensus on the diagnostic criteria of irAE due to its versatile presentation, despite the widespread prescription of ICIs in multiple cancers.

The diagnosis of irAE relies on the presence of a typical manifestation of commonly involved organs in the context of treatment with ICIs, which excludes other possible etiology. That said, while all changes in these end-organ functions as observed following the use of ICIs should suspected as a treatment-related effect, no causation could be directly inferred from the temporality; moreover, rare presentations of irAE and the diagnosis of such conditions remained challenging. As such, studies on the immunologic deviation and pathophysiology of irAE, as evidenced by biopsies and immunohistochemistry staining, are warranted to develop guidelines for the diagnosis of irAE.

## Treatment modalities of irAEs

The management of irAEs depends on the severity, the involved organs, and the tumor response to ICIs. Corticosteroids are used as the primary treatment for irAE, and most patients respond well to corticosteroids (10). Recent studies showed that immune modulatory treatments for irAEs were effective and did not compromise the objective response rate and progression-free survival of cancers (11). In particular, pulse steroid therapy demonstrated a neurological improvement rate of 90% in a study with 11 immune-related encephalitis cases, it was therefore recommended for the initial treatment of immune-related encephalitis in the guideline (12) and had been used to treat ICI-associated interstitial nephritis and pneumonitis (13).

Intravenous immunoglobulin (IVIg), biological agents, such as infliximab and rituximab, and plasmapheresis, have been used to manage steroid-refractory cases of irAEs (14). Treatment response to these agents varied based on the organ involved and the immune response triggered by ICIs. With evidence on immunohistochemistry staining, the immune response mounted by ICIs against the affected organ could be revealed, which may suggest suitable immune modulators used to treat steroid-refractory cases of irAE.

## The presence of irAEs and continuation of ICI treatments

Clinicians are primarily concerned about the continuation of ICI treatment. Both pembrolizumab and nivolumab have a mean elimination half-life of roughly 26 days, while the effects of programmed cell death protein 1 (PD-1) inhibitors last longer. Although some studies suggested that the presence of non-fatal irAEs may indicate better anti-neoplastic treatment response and survival outcomes (15), significant irAEs require extreme cautions when it comes to the decision of reintroducing immunotherapy, for which the management of irAEs and the judgment of whether to continue ICI treatments should be decided by a multidisciplinary tumor board that involves medical oncologists and specialists including dermatologists, ophthalmologists, cardiologists, pulmonologists, gastroenterologists, and endocrinologists.

## Summary

Real-world observations on the safety of ICIs provide evidence for the epidemiology, clinical presentations, and prognosis of irAEs. The assessment and management of irAE require multidisciplinary collaborations, so as the diagnostic and treatment guidelines for irAE. This Research Topic, with valuable records of clinical experience in anti-neoplastic agent-related adverse events, reflects the global awareness of adverse drug reactions in medical oncology.

## References

[1] Ma KS Saeed HN Chodosh J Wang CW Chung YC Wei LC Ocular manifestations of anti-neoplastic immune checkpoint inhibitor-associated Stevens-Johnson syndrome/toxic epidermal necrolysis in cancer patients. Ocul Surf. (2021) 22:47–50. 10.1016/j.jtos.2021.06.010 34216790

[2] Chiang CH Chiang CH Ma KS Hsia YP Lee YW Wu HR The incidence and risk of cardiovascular events associated with immune checkpoint inhibitors in Asian populations. Jpn J Clin Oncol. (2022) 52(12):1389–98. 10.1093/jjco/hyac150 36208180 PMC9721460

[3] Schneider BJ Naidoo J Santomasso BD Lacchetti C Adkins S Anadkat M Management of immune-related adverse events in patients treated with ICI therapy: ASCO guideline update. J Clin Oncol. (2021) 39(36):4073–126. 10.1200/JCO.21.01440 34724392

[4] Haanen JBAG Carbonnel F Robert C Kerr KM Peters S Larkin J Management of toxicities from immunotherapy: ESMO Clinical Practice Guidelines for diagnosis, treatment and follow-up. Ann Oncol. (2017) 28(suppl_4):iv119–42. 10.1093/annonc/mdx225 28881921

[5] Pirozzi F Poto R Aran L Cuomo A Galdiero MR Spadaro G Cardiovascular toxicity of immune checkpoint inhibitors: clinical risk factors. Curr Oncol Rep. (2021) 23(2):13. 10.1007/s11912-020-01002-w 33415405 PMC7790474

[6] Abdel-Wahab N Shah M Lopez-Olivo MA Suarez-Almazor ME. Use of ICIs in the treatment of patients with cancer and preexisting autoimmune disease: a systematic review. Ann Intern Med. (2018) 168(2):121–30. 10.7326/M17-2073 29297009

[7] Redelman-Sidi G Michielin O Cervera C Ribi C Aguado JM Fernandez-Ruiz M ESCMID Study Group for Infections in Compromised Hosts (ESGICH) Consensus Document on the safety of targeted and biological therapies: an infectious diseases perspective (ICIs, cell adhesion inhibitors, sphingosine-1-phosphate receptor modulators and proteasome inhibitors). Clin Microbiol Infect. (2018) 24(Suppl 2(Suppl 2)):S95–S107. 10.1016/j.cmi.2018.01.030 29427804 PMC5971148

[8] Ma KS Chung WH Hsueh YJ Chen SY Tokunaga K Kinoshita S Human leucocyte antigen association of patients with Stevens-Johnson syndrome/toxic epidermal necrolysis with severe ocular complications in Han Chinese. Br J Ophthalmol. (2022) 106(5):610–5. 10.1136/bjophthalmol-2020-317105 33441319

[9] Ma KS Wei JC Chung WH. Correspondence to “Hypersensitivity reactions with allopurinol and febuxostat: a study using the Medicare claims data”. Ann Rheum Dis. (2022) 81(6):e107. 10.1136/annrheumdis-2020-218090 32561605

[10] Brahmer JR Lacchetti C Schneider BJ Atkins MB Brassil KJ Caterino JM Management of immune-related adverse events in patients treated with ICI therapy: American society of clinical oncology clinical practice guideline. J Clin Oncol. (2018) 36(17):1714–68. 10.1200/JCO.2017.77.6385 29442540 PMC6481621

[11] Weber JS Hodi FS Wolchok JD Topalian SL Schadendorf D Larkin J Safety profile of nivolumab monotherapy: a pooled analysis of patients with advanced melanoma. J Clin Oncol. (2017) 35(7):785–92. 10.1200/JCO.2015.66.1389 28068177

[12] Brahmer JR Abu-Sbeih H Ascierto PA Brufsky J Cappelli LC Cortazar FB Society for Immunotherapy of Cancer (SITC) clinical practice guideline on ICI-related adverse events. J Immunother Cancer. (2021) 9(6):e002435. 10.1136/jitc-2021-002435 34172516 PMC8237720

[13] Manohar S Ghamrawi R Chengappa M Goksu BNB Kottschade L Finnes H Acute interstitial nephritis and checkpoint inhibitor therapy: single center experience of management and drug rechallenge. Kidney360. (2020) 1(1):16–24. 10.34067/KID.0000152019 35372854 PMC8808482

[14] Asdourian MS Shah N Jacoby TV Reynolds KL Chen ST. Association of bullous pemphigoid with ICI therapy in patients with cancer: a systematic review. JAMA Dermatol. (2022) 158(8):933–41. 10.1001/jamadermatol.2022.1624 35612829

[15] Nelson CA Singer S Chen T Puleo AE Lian CG Wei EX Bullous pemphigoid after anti-programmed death-1 therapy: a retrospective case-control study evaluating impact on tumor response and survival outcomes. J Am Acad Dermatol. (2022) 87(6):1400–2. 10.1016/j.jaad.2019.12.068 31931083

